# The role of a multidisciplinary approach in the early and differential diagnosis of inflammatory bowel disease–related spondyloarthritis: insights from a cross-sectional study

**DOI:** 10.3389/fmed.2026.1824694

**Published:** 2026-05-12

**Authors:** Alberto Floris, Leonardo Sichi, Agnese Favale, Marcella Falconi, Andrea Pace, Maria Maddalena Angioni, Angelo Italia, Raffaela Piras, Federica Olla, Francesca Onnis, Matteo Piga, Massimo Claudio Fantini, Sara Onali, Alberto Cauli

**Affiliations:** 1 Dipartimento di Scienze Mediche e Sanità Pubblica, Università di Cagliari, Monserrato, Italy; 2 SC di Reumatologia, Azienda Ospedaliero Universitaria di Cagliari, Monserrato, Italy; 3 SC di Gastroenterologia, Azienda Ospedaliero Universitaria di Cagliari, Monserrato, Italy

**Keywords:** differential diagnosis, early diagnosis, IBD-SpA, inflammatory bowel disease, multidisciplinary approach, spondyloarthritis

## Abstract

**Objectives:**

Inflammatory bowel disease-related spondyloarthritis (IBD-SpA) commonly complicates IBD. Early diagnosis is essential; however, it is often delayed due to clinical heterogeneity and the lack of specific diagnostic criteria. This study aimed to evaluate the outcomes of close collaboration between gastroenterologists and rheumatologists in the classification of the musculoskeletal (MSK) manifestations in IBD patients, focusing on the early diagnosis of IBD-SpA and its differentiation from other conditions.

**Methods:**

Consecutive IBD outpatients underwent a comprehensive rheumatologic evaluation during a joint rheumatologist–gastroenterologist consultation, including the DETection of Arthritis in Inflammatory boweL diseases (DETAIL) screening questionnaire, and, when appropriate, laboratory and imaging investigations. Suspected IBD-SpA cases were classified as newly diagnosed IBD-SpA, having an alternative arthropathy diagnosis, or having non-specific arthralgias, and their characteristics were analyzed.

**Results:**

A total of 605 IBD patients were evaluated. At the first assessment, 81 patients had a pre-existing diagnosis of SpA and 117 patients of suspected IBD-SpA cases were identified. Among the latter assessment, 18 (15%) were confirmed as new IBD-SpA diagnoses, resulting in a 22% relative increase in the total number of SpA cases and raising the overall prevalence from 13.4 to 16.4%. Patients with axial involvement experienced a longer diagnostic delay (mean 12.4 vs. 2.9 years, *p* = 0.035). Of the new IBD-SpA diagnoses, 72% resulted in treatment modifications jointly decided by the gastroenterologist and rheumatologist. However, 85% of suspected IBD-SpA cases were ultimately excluded, with 60% classified as other arthropathies (primary osteoarthritis and fibromyalgia) and 25% as non-specific arthralgias. The swollen joint count was the only significant predictor of a new IBD-SpA diagnosis (adjOR 5.70, *p* < 0.001), with a trend toward significance for fecal calprotectin levels ≥200 μg/g (adjOR 2.88, *p* = 0.070).

**Conclusion:**

The multidisciplinary approach enables early and accurate diagnosis of IBD-SpA, minimizing the risk of misclassification of musculoskeletal manifestations and unwarranted treatment changes.

## 1 Introduction

The term spondylarthritis (SpA) refers to a group of chronic inflammatory diseases that primarily affect the axial skeleton, peripheral joints, and entheses and may be associated with extra-articular manifestations including psoriasis, uveitis, and inflammatory bowel disease (IBD) (1). Within the spectrum of SpA, the forms that are associated with IBD, including Crohn’s disease (CD) and ulcerative colitis (UC), are commonly classified as IBD-related spondylarthritis (IBD-SpA) (2). IBD-SpA is characterized by a wide clinical heterogeneity of musculoskeletal (MSK) manifestations, which may develop in patients with established IBD or may even precede the onset of overt gastrointestinal (GI) symptoms (3). The estimated prevalence of IBD-SpA in IBD patients ranges from 4 to 23%, based on the evaluated cohorts, their geographic origin, and the protocol applied for SpA cases identification (3).

Early diagnosis is crucial in the management of all SpA patients, as a longer diagnostic delay is associated with higher rates of structural damage progression, disability, and impaired quality of life (4). However, early SpA diagnosis remains one of the main challenges. Indeed, the diagnostic delay still averages around 7 years in axial SpA and 11 months in primarily peripheral arthritis, such as psoriatic arthritis (PsA) (5, 6). With regard to IBD-SpA, early and accurate diagnosis can be particularly challenging due to the heterogeneity of MSK and GI manifestations, the non-specific nature of joint symptoms, and the masking effects of IBD treatments–particularly corticosteroids and biologic agents–which may together contribute to underdiagnosis or delayed referral (7). Moreover, there are no specific classification criteria for IBD-SpA. Indeed, the Assessment of Spondyloarthritis International Society (ASAS) criteria may help identify SpA; however, their specific validity in the diagnosis of IBD-SpA remains unproven (8). Disease-specific factors—such as lower HLA-B27 prevalence, limited use of non-steroidal anti-inflammatory drugs (NSAIDs), and non-specific C-reactive protein (CRP) elevation—may impact the accuracy of these criteria (9).

Present recommendations emphasize the role of a multidisciplinary approach involving both rheumatologists and gastroenterologists for the management of IBD-SpA (10, 11), through the implementation of strategies, such as appropriate screening for SpA, in all IBD patients and vice versa, a combined clinical assessment of both MSK and GI domains, and a shared treatment approach (12). Indeed, a major challenge in diagnosing IBD-SpA lies in distinguishing the disease from other arthropathies that can mimic SpA. The presence of IBD does not exclude the possibility of concomitant and often more widespread joint disorders. Therefore, MSK manifestations in IBD patients should not be automatically interpreted as indicative of SpA, as this may lead to misclassification and overtreatment.

Furthermore, a relevant entity in the differential diagnosis is non-specific arthralgias—defined as joint pain not attributable to SpA or other specific arthropathies. These cases represent an intriguing clinical scenario, as they may constitute a prodromal or very early form of SpA and could become targets for future interventional strategies. However, while an increasing body of evidence supports this hypothesis in the context of PsA, corresponding data in IBD-SpA remain limited (13).

This study aimed to assess the outcomes, advances, and challenges of a structured collaboration between gastroenterologists and rheumatologists within a shared outpatient clinic for the management of IBD patients, particularly in the identification of early or undiagnosed IBD-SpA, and its differentiation from other mimicking conditions and non-specific arthralgias.

## 2 Patients and methods

### 2.1 Patients and study design

This is a preliminary and cross-sectional analysis of the DIAMANTE (Early Diagnosis of Enteropathic Spondyloarthritis in a Cohort of Patients with Inflammatory Bowel Disease) study, an ongoing project aimed at prospectively identifying the prevalence, predictors, and features of IBD-SpA within the framework of a structured collaboration between gastroenterologists and rheumatologists.

In this study, a cohort of consecutive IBD patients was recruited from the IBD clinic of the Gastroenterology Unit of the University Hospital of Cagliari, according to the following inclusion criteria: (a) age ≥ 18 years; (b) diagnosis of IBD according to European Crohn’s and Colitis Organisation (ECCO) criteria (14, 15), and (c) provision of informed consent for study participation and completion of the planned rheumatological assessment. No specific exclusion criteria were defined.

At enrollment, after signing informed consent, all patients underwent an in-depth rheumatologic evaluation during their routine IBD visit by the SpA clinic team of the Rheumatology Unit of the same Institution, composed of three experienced rheumatologists and two rheumatology residents.

The following data were recorded for all patients: demographics, smoking habits, family history of IBD and SpA, family or personal history of psoriasis and uveitis, the IBD phenotype and disease extent or location according to the Montreal classification. IBD clinical disease activity was assessed using the Harvey–Bradshaw Index (HBI) for CD (16), and the partial Mayo score (pMAYO) for UC (17), along with biomarkers such as C-reactive protein (CRP) and fecal calprotectin (FC) levels. Moreover, the selection of the HBI and partial Mayo scores was guided with the aim of contextualizing the study results within real-world clinical practice. Indeed, although not fully validated, these indices are extensively used in routine care due to their practicality and reliability. The endoscopic disease activity was assessed using the Simple Endoscopic Score for CD (SES-CD) (18) and the Mayo Endoscopic Score for UC (19).

### 2.2 Rheumatologic evaluation

The rheumatologic evaluation was performed in a real-world clinical setting, with the rheumatologist aware of the findings of the preceding gastroenterological assessment, according to the following protocol:

(a) A comprehensive review of existing and past medical history, alongside demographic and clinical data, with particular attention to MSK symptoms.
(b) A physical examination, including a systematic evaluation of the 66 swollen joint count and 68 tender peripheral joint count, presence of active dactylitis, enthesitis, tender points (as defined by the 1990 fibromyalgia classification criteria) (20), and other clinical signs indicative of coexisting arthropathies (such as Heberden’s or Bouchard’s nodes, rheumatoid nodules, and tophi) or alternative rheumatologic conditions potentially underlying MSK symptoms.
(c) Laboratory investigations, including CRP, erythrocyte sedimentation rate (ESR), and, if appropriate, rheumatoid factor, anti-cyclic citrullinated peptide antibodies, and HLA-B27.
(d) Administration of the DETection of Arthritis in Inflammatory boweL diseases (DETAIL) questionnaire at the start of the visit (21). This self-administered, six-item questionnaire assesses signs and symptoms potentially attributable to IBD-SpA, scoring 1 point per item present. A total score of ≥3 has been validated as a threshold for identifying suspected IBD-SpA cases (22).
(e) Imaging investigations, including X-ray, magnetic resonance imaging (MRI), and ultrasound (US), were performed when clinically indicated. In line with the overall aim of the study—to evaluate a multidisciplinary approach in a real-life setting—imaging was not based on a predefined protocol but was instead undertaken on a case-by-case basis, according to the rheumatologist’s clinical judgment and the European Alliance of Associations for Rheumatology (EULAR) recommendations (23). In particular, spine and sacroiliac joint X-rays and MRI were performed in patients with low back pain, particularly in the presence of inflammatory features, to support the differential diagnosis of sacroiliitis and/or spondylitis and to distinguish these conditions from other causes, such as spondylosis with intervertebral disc degeneration. Peripheral joint X-rays were primarily used to aid in the differential diagnosis of osteoarthritis. US played a crucial role in equivocal cases of synovitis and was mandatory to confirm or exclude all clinically suspected cases of enthesitis.

### 2.3 Definitions of rheumatologic classification outcomes

Based on the study objectives and the results of the gastro-rheumatologic evaluation, patients were classified into the following groups:

(i) *Pre-existing diagnosis of SpA*: The SpA diagnoses were retrospectively and critically reviewed and were confirmed if formally made by a rheumatologist fulfilling the ASAS classification criteria (1). For all confirmed pre-existing IBD-SpA, the following clinical criteria were retrospectively recorded: presence of inflammatory articular manifestations, personal or family history of psoriasis, dactylitis, new juxta-articular bone formation on X-rays of hands or feet, and negative rheumatoid factor.
(ii) *Suspected IBD-SpA*: Patients without a previous SpA diagnosis but with clinical suspicion based on rheumatologist evaluation and/or a DETAIL score ≥3 were included. After a complete rheumatologic workup, these cases were classified as follows:

- *New diagnosis of IBD-SpA*: Diagnosis established according to ASAS criteria following the rheumatologic assessment (1). For all patients with newly diagnosed IBD-SpA, additional data were collected: articular pattern, patient global assessment (PtGA), pain measured by visual analog scale (VAS), and any treatment changes following the diagnosis.
- *Alternative diagnosis*: Another diagnosis explaining MSK symptoms, based on clinical examination, US, and other investigations. When applicable, diagnoses were made using validated criteria, such as fibromyalgia, rheumatoid arthritis, and gout (20, 24–26).
- *Non-specific arthralgia*: Arthralgia not explained by IBD-SpA or another rheumatologic condition.

If a patient received both a new diagnosis of IBD-SpA and another MSK disease, they were classified under “new diagnosis of IBD-SpA” with the second condition recorded separately as an overlapping condition.

### 2.4 Statistical analysis

Categorical variables are reported as absolute numbers and frequencies (%). Continuous variables are reported as mean ± standard deviation (±SD). The univariate analysis was performed using the chi-square and Fisher’s exact test or the Mann–Whitney test to identify demographic and clinical variables associated with the new diagnosis of PsA within the group of patients with suspected PsA. The multivariate logistic regression analysis was performed to confirm the independent associations identified in the univariate analysis. The effect size for such an association was expressed as odds ratios (ORs) with a 95% confidence interval (CI). Two separate sub-analyses were performed, distinguishing patients with UC and CD, and including disease-specific features (extent, localization, behavior, clinical, and endoscopic activity indices) as potential factors associated with a new IBD-SpA diagnosis. Furthermore, the inferential statistical analysis using the Mann–Whitney test was performed to compare the average diagnostic delay in patients with and without axial involvement. Statistical significance was set at a *p*-value of < 0.05.

All analyses were carried out using SPSS (version 24.0, IBM^©^).

## 3 Results

### 3.1 Patients

A total of 605 consecutive IBD patients were recruited: 238 (39.3%) patients had Crohn’s disease (CD), 340 (56.2%) had ulcerative colitis (UC), and 27 (4.5%) had IBD unclassified (IBD-U). Among the patients, 322 (53.3%) were women, and the mean (±SD) age at enrollment was 47.5 (±12.8) years. Demographic and clinical details on the whole study cohort are reported in Table 1.

**Table 1:** Baseline features of the entire cohort.

Variable	(*n* = 605)
Men, *n* (%)	281 (46.8%)
Age, mean (SD) years	51.1 (15.6)
IBD disease duration, mean (SD) years	15.5 (10.3)
BMI, mean (SD)	23.9 (4.4)
IBD classification	
Ulcerative colitis, *n* (%)	340 (56.2%)
*Localization*	
E1	86
E2	90
E3	157
*Partial Mayo score, mean (SD) endoscopic Mayo*	0.7 (1.6)
S0	82
S1	25
S2	40
S3	25
Crohn’s disease, *n* (%)	238 (39.3%)
*Localization*	
L1, *n*	62
L2, *n*	31
L3, *n*	138
L4, *n*	0
*Behavior*	
B1, *n*	135
B2, *n*	72
B3, *n*	22
Perineal disease, *n*	58
IBD undifferentiated, *n* (%)	27 (4.5%)
Family history	
IBD, *n* (%)	85 (14.3%)
Psoriasis, *n* (%)	109 (18.1%)
Personal history	
SpA, *n* (%)	81 (13.4%)
Psoriasis, *n* (%)	60 (10.0%)
Uveitis, *n* (%)	17 (2.8%)
Nodosum erythema, *n* (%)	13 (2.2%)
Gangrenous Pyoderma, *n* (%)	3 (0.5%)
DETAIL score, mean (SD) years	1.1 (1.6)
Current treatment	
Systemic glucocorticoids, *n* (%)	41 (7.3%)
Mesalazine, *n* (%)	392 (64.8%)
*Conventional IS*	37
Azathioprine, *n*	17
Sulfasalazine, *n*	15
Methotrexate, *n*	5
*Advanced targeted drugs, n (%)*	262 (43.3%)
TNFi, *n*	161
IL-12/23i and IL-23i, *n*	47
Vedolizumab, *n*	25
JAKi, *n*	31

SD, standard deviation; BMI, body mass index; IBD, inflammatory bowel disease; SpA, spondyloarthritis; DETAIL, DETection of Arthritis in Inflammatory boweL diseases questionnaire; TNFi, tumor necrosis factor inhibitor; IL12/23i, interleukin 12/23 inhibitor; IL-23i, interleukin-23 inhibitor; JAK, Janus-Kinase inhibitor.

### 3.2 Previous diagnoses of IBD-SpA and other chronic inflammatory arthropathy

**Figure 1:**
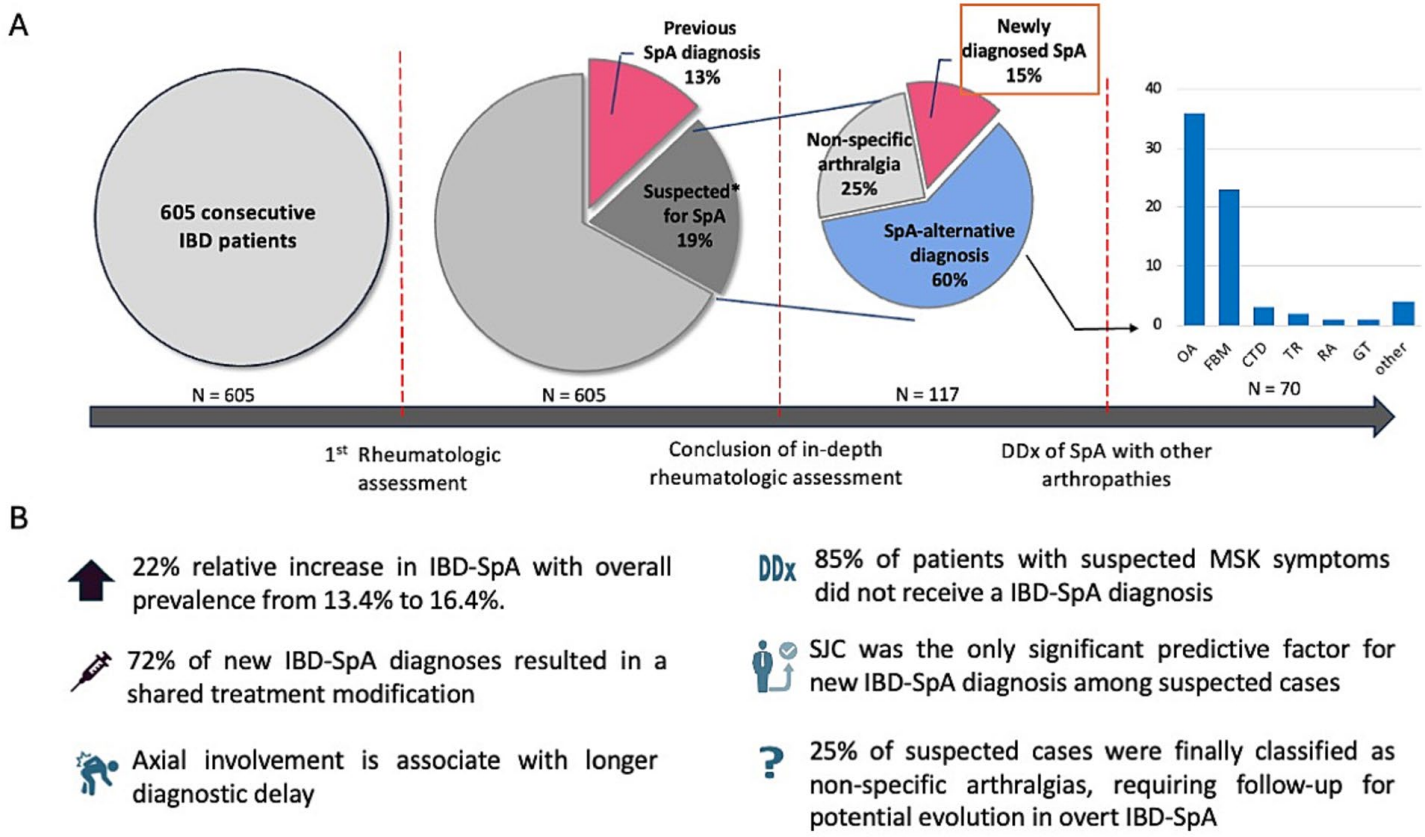
Graphical representation of the sequential phases of the differential diagnostic work-up for IBD-SpA **(A)** and summary of the key findings **(B)**. IBD, inflammatory bowel disease; SpA, spondyloarthritis; DDx, differential diagnosis; OA, osteoarthritis; FBM, fibromyalgia; CTD, connective tissue disease; RA, rheumatoid arthritis; GT, gout; SJC, swollen joint count; FC, fecal calprotectin.

A pre-existing diagnosis of SpA was recorded in 81 (13.4%) patients (Figure 1). They included 72 cases classified as IBD-SpA, 6 cases classified as PsA with IBD, and 3 cases who had a previous diagnosis of rheumatoid arthritis that was reclassified as IBD-SpA after the rheumatologic assessment based on an in-depth review of clinical, serological, and imaging features. The mean SpA disease duration was 14.6 (±11.7) years. In total, 54 (66.7%) patients had peripheral joint involvement and 44 (54.3%) had axial involvement, with a 17.5% overlap rate. The onset of IBD-SpA occurred after the onset of IBD in 43.2% of cases; it preceded the onset of IBD in 34.6% of cases; and both conditions developed at nearly the same time in 22.2% of cases. Details on patients with previous IBD-SpA diagnosis are reported in the Supplementary materials. In the studied IBD cohort, two patients had a previous diagnosis of rheumatoid arthritis, which was confirmed after the critical rheumatologic review at the study visit.

### 3.3 Patients with suspected IBD-SpA

After rheumatological assessment, 117 (19.3%) cases were classified as suspected IBD-SpA (Figure 1): 29 (24.8%) based on both the rheumatologist’s judgment and the DETAIL screening questionnaire, and 39 (33.3%) and 49 (41.9%) were classified solely based on the rheumatological evaluation or DETAIL questionnaire positivity, respectively.

### 3.4 New diagnoses of IBD-SpA

Of the 117 suspected IBD-SpA cases, 18 (15.4%) received a final diagnosis of IBD-SpA (Figure 1), with an increase in the overall prevalence of IBD-SpA in the whole IBD cohort from 13.4 to 16.4% (relative increase of 22.2%). Within the group of 18 patients with a new IBD-SpA diagnosis, 6 (33.3%) were men and the mean (±SD) IBD duration was 15.6 (±10.8) years. Peripheral involvement was recorded in 16 (88.9%) patients. Axial involvement was recorded in 5 (27.8%) patients: 3 were classified as non-radiographic axial SpA (MRI positive and X-ray negative), and 2 as radiographic axial SpA (both MRI and X-ray positive). The mean diagnostic delay from the onset of MSK symptoms to IBD-SpA was 3.1 (±3.4) years, with 8 (44.4%) patients receiving the IBD-SpA diagnosis within 1 year, and 3 (16.7%) patients between 1 and 2 years from symptom onset. A significantly higher diagnostic delay was recorded in patients with axial involvement (mean ± SD = 12.4 ± 10.2 vs. 2.9 ± 2.9, *p* = 0.035). None of the patients with a new diagnosis showed positivity for rheumatoid factor or anti-citrullinated peptide antibodies. Two patients were HLA-B27 positive (subtypes B*2705 and B*2709, respectively); however, neither presented with evidence of axial involvement on X-ray or MRI. Further details on demographic and clinical characteristics of patients with a new diagnosis of IBD-SpA are reported in Table 2.

**Table 2:** Demographic and clinical features of patients with a new IBD-SpA diagnosis, alternative diagnosis, and non-specific arthralgias.

Variable	New IBD-SpA (*n* = 18)	Alternative diagnosis (*n* = 70)	Non-specific arthralgias (*n* = 29)
Men, *n* (%)	6 (33.3)	22 (31.4)	13 (44.8)
Age, mean (SD) years	51.8 (16.6)	54.2 (11.5)	44.9 (12.3)
BMI, mean (SD)	23.5 (10.1)	24.1 (5.7)	23.1 (4.1)
IBD duration, mean (SD) years	15.6 (10.8)	17.8 (9.1)	12.3
IBD classification			
UC, *n* (%)	10 (55.6)	40 (57.1)	11 (37.9)
CD, *n* (%)	7 (38.9)	25 (35.7)	18 (62.1)
Other, *n* (%)	1 (5.6)	5 (7.2)	0
Fecal calprotectin >200 μg/g, *n* (%)	8 (44.4)	10 (16.4)	4 (16.7)
Extraintestinal manifestation			
Uveitis, *n* (%)	0	1 (1.4)	1 (3.4)
Psoriasis, *n* (%)	1 (5.6)	9 (13.0)	4 (13.8)
Ongoing treatment			
Systemic glucocorticoids, *n* (%)	3 (17.6)	6 (9.1)	4 (15.4)
Conventional drugs			
*SLZ, n (%)*	0	4 (5.7)	0
AZA, *n* (%)	0	1 (1.4)	0
MTX, *n* (%)	0	0	0
*Targeted advanced drugs, n (%)*	10 (55.6)	32 (45.7)	13 (44.8)
TNFi, *n* (%)	5 (27.8)	18 (25.7)	10 (34.5)
IL12/23i and IL23i, *n* (%)	3 (16.3)	6 (8.6)	3 (10.3)
Vedolizumab, *n* (%)	2 (11.1)	7 (10.0)	0
JAKi, *n* (%)	0	2 (2.9)	0
DETAIL score, mean (SD)	1.8 (1.4)	3.2 (1.6)	3.1 (1.4)
Rheumatologic examination			
Tender joint count, mean (SD)	1.8 (2.0)	1.2 (81.2)	0.9 (1.7)
Swollen joint count, mean (SD)	1.3 (0.2)	0.1 (0.3)	0.2 (0.8)
IBD-SpA disease duration, mean (SD) years	4.9 (7.1)	–	–
IBD-SpA diagnostic delay, mean (SD) years	3.1 (3.4)	–	–
IBD-SpA pattern			
*Peripheral arthritis, n (%)*	16 (88.9)	–	–
Oligoarthritis, *n* (%)	14 (77.8)	–	–
Polyarthritis, *n* (%)	2 (11.1)	–	–
*Axial involvement, n (%)*	5 (27.8)	–	–
Enthesitis, *n* (%)	2 (11.1)	–	–
Dactylitis, *n* (%)	3 (16.7)	–	–

SD, standard deviation; BMI, body mass index; IBD, inflammatory bowel disease; SpA, spondyloarthritis; DETAIL, DETection of Arthritis in Inflammatory boweL diseases questionnaire; SLZ, sulfasalazine; AZA, azatioprina; MTX, methotrexate; TNFi, tumor-necrosis-factor inhibitor; IL12/23i, interleukin 12/23 inhibitor; IL-23i, interleukin-23 inhibitor; JAK, Janus-Kinase inhibitor.

All cases of patients with a new IBD-SpA diagnosis were jointly reviewed by the rheumatologist and the gastroenterologist for possible treatment optimization. In 13 cases (72.2%), therapy was modified to achieve better overall disease control, addressing both articular and intestinal disease. In the remaining cases, although no immediate treatment change was implemented, closer follow-up was scheduled.

### 3.5 Alternative diagnoses

Among the 117 IBD-SpA suspected cases, 70 (59.8%) received an alternative diagnosis explaining their MSK symptoms. In particular, among the alternative diagnosis, 36 (51.4%) cases were of osteoarthritis, 23 (32.9%) cases of fibromyalgia, 3 (4.3%) cases of connective tissue disease-related arthritis, 1 (1.4) case of chronic mechanical tendinopathy, 1 (1.4%) case of rheumatoid arthritis with anti-citrullinated peptide antibody, 1 (1.4%) case of complex regional pain syndrome (CRPS), 1 (1.4%) case of gout, 1 (1.4%) case of polymyalgia rheumatica, 1 (1.4%) case of diffuse idiopathic skeletal hyperostosis (DISH), 1 (1.4%) case of post-traumatic pain (Figure 1). Details of this subgroup of patients are reported in Table 2.

All patients received specific recommendations regarding follow-up, pharmacological, and non-pharmacological interventions to manage their newly diagnosed condition.

### 3.6 Non-specific arthralgia

Among patients suspected of having SpA, 29/117 (24.8%) were finally classified as having non-specific arthralgias (Figure 1), not attributable to IBD-SpA or other arthropathies. The mean duration of symptoms in this group was 4.8 ± 4.1 years, and 11 patients (73.3%) reported symptoms lasting more than 1 year. All patients were informed regarding the importance of surveillance and follow-up for the possible evolution to SpA. Details on the demographic, clinical, and therapeutic characteristics of this subgroup of patients are reported in Table 2.

### 3.7 Factors associated with new IBD-SpA diagnosis

In the univariate analysis, newly IBD-SpA diagnosed patients had significantly higher prevalence of FC levels of >200 mcg/g (44.4% vs. 18.2%, *p* = 0.006), and higher values of the DETAIL score (mean ± SD = 1.8 ± 1.4 vs. 0.8 ± 1.4, *p* < 0.001), as well as higher tender (1.8 ± 2.0 vs. 0.3 ± 1.0, *p* < 0.001) and swollen joint counts (1.3 ± 0.2 vs. 0.0 ± 0.2, *p* < 0.001), compared with patients in whom IBD-SpA was excluded. In the multivariate analysis, only the swollen joint count was confirmed to be independently associated with IBD-SpA diagnosis (adjOR 5.70, 95% CI = 2.18–14.92, *p* < 0.001). A trend toward statistical significance was recorded for FC levels >200 mcg/g (adjOR 2.88, 95% CI = 0.92–9.07, *p* = 0.070) (Table 3). No other demographic, clinical, or treatment-related factors were found to influence the likelihood of a new SpA diagnosis. Regarding treatment in a further analysis in which vedolizumab was evaluated separately from other advanced therapies, it was not associated with an increased risk of new-onset SpA (*p* = 0.185).

**Table 3:** Predictors of new IBD-SpA diagnosis.

	Univariate analysis			Multivariate analysis	
Variable	New IBD-SpA diagnosis (*n* = 18)	Excluded IBD-SpA diagnosis (*n* = 504)	*p*	AdjOR (95% CI)	*p*
Men, *n* (%)	6 (33.3)	240 (47.6)	0.233		
Age, mean (SD) years	51.8 (16.6)	50.9 (16.0)	0.767		
BMI, mean (SD)	23.5 (10.1)	23.9 (4.4)	0.817		
IBD duration, mean (SD) years	15.6 (10.8)	15.0 (10.1)	0.798		
IBD classification			0.454		
UC, *n* (%)	10 (55.6)	182 (36.1)			
CD, *n* (%)	7 (38.9)	299 (59.3)			
Other, *n* (%)	1 (5.6)	23 (4.6)			
Fecal calprotectin >200 μg/g, *n* (%)	8 (44.4)	77 (18.2)	0.006	2.88 (0.92–9.07)	0.070
Family history of SpA, *n* (%)	1 (5.6)	57 (11.5)	0.708		
Extraintestinal manifestation					
Uveitis, *n* (%)	0	8 (1.6)	1.00		
Psoriasis, *n* (%)	1 (5.6)	37 (7.4)	1.00		
Ongoing treatment					
Systemic glucocorticoids, *n* (%)	3 (17.6)	34 (7.2)	0.131		
Targeted advanced drugs, *n* (%)	10 (55.6)	192 (38.7)	0.218		
DETAIL score, mean (SD)	1.8 (1.4)	0.8 (1.4)	<0.001	1.17 (0.85–1.61)	0.328
DETAIL score ≥3, *n* (%)	5 (27.8)	73 (14.5)	0.120		
Rheumatologic examination					
Tender joint count, mean (SD)	1.8 (2.0)	0.3 (1.0)	<0.001	1.05 (0.74–1.47)	0.794
Swollen joint count, mean (SD)	1.3 (0.2)	0.0 (0.2)	<0.001	5.70 (2.18–14.92)	<0.001

SD, standard deviation; BMI, body mass index; IBD, inflammatory bowel disease; SpA, spondyloarthritis; DETAIL, DETetection of Arthritis in Inflammatory boweL diseases questionnaire; adjOR, adjusted odds ratio; CI, confidence interval.

In the sub-analyses distinguishing patients with CD and UC, and including disease-specific characteristics (such as extent, location, behavior, and clinical and endoscopic activity scores) as potential predictors of a new IBD-SpA diagnosis, results were consistent with those of the whole-cohort analysis, and no additional significant associations emerged (data not shown).

### 3.8 Role of MRI and US in the real-life clinical setting

Back pain was reported by 78 (67%) patients with suspected SpA, but only 21 (18%) patients met the ASAS criteria for inflammatory back pain (IBP). Spine and sacroiliac joints X-ray and MRI were performed in all patients with IBP, confirming axial involvement in 19 (24.4%) cases. All confirmed cases had evidence of active sacroiliitis and/or spondylitis on MRI according to the ASAS criteria and 2 also had X-ray evidence of sacroiliitis and spondylitis. Among 57 patients with non-inflammatory low back pain, all underwent spinal and sacroiliac joint X-rays, while an MRI was performed in one-third of cases. This finding enabled attribution of such axial symptoms to osteoarthritis in 50.9% of cases, fibromyalgia in 20.1%, and non-specific causes in 20.0%.

US was conducted in 68 (58.1%) patients with suspected IBD-SpA and peripheral symptoms. Synovitis and/or tenosynovitis were confirmed in all patients with a clinical diagnosis of peripheral arthritis. Moreover, the US revealed enthesal involvement in 2 (2.9%) patients with SpA. On the other hand, US played a critical role in excluding enthesitis in 12 (52.2%) of the 23 patients who were finally diagnosed with fibromyalgia, despite the presence of typical tender points on physical examination. Furthermore, the US supported the differential diagnosis of microcrystalline arthropathy in one patient.

## 4 Discussion

This study provides original and clinically relevant data regarding the challenges of early recognition and differential diagnosis of SpA in IBD patients, emphasizing the essential value of a close collaboration between gastroenterologists and rheumatologists.

As a first key finding, this study showed that implementing a multidisciplinary protocol led to a 22% relative increase in SpA diagnoses among IBD patients, raising overall prevalence from 13.4 to 16.4%. Of interest, this increase resulted from the identification of both early and established IBD-SpA cases and was associated with longer diagnostic delay among patients with axial involvement. Similarly, in an IBD cohort study, Luchetti et al. reported a mean time from symptom onset to SpA diagnosis of approximately 4 years, with a significantly longer diagnostic delay in patients with axial involvement, compared with those with isolated peripheral disease (5.5 vs. 2.3 years, *p* = 0.003). In particular, this study clearly shows that recognizing and characterizing IBP is essential for identifying suspected axSpA in patients with IBP; however, this study also highlights its low positive predictive value, given that approximately one-fifth of patients meeting IBP criteria demonstrated sacroiliitis and/or spondylitis on MRI or X-ray. From a practical point of view, this finding underscores the importance of systematically searching for IBP in IBD patients and simultaneously the need for imaging confirmation to improve sensitivity in detecting axSpA, while minimizing the risk of overdiagnosis. Supporting the clinical relevance of increased sensitivity in diagnosing SpA among patients with IBD, this study shows that enhanced detection does not simply inflate prevalence estimates but, more importantly, translates into more comprehensive and patient-centered disease management. In approximately three-quarters of cases, a new SpA diagnosis led to a shared modification of treatment, while in the remaining cases, it resulted in at least an adjustment of follow-up strategies. The integration of early and accurate diagnosis through a multidisciplinary clinical evaluation, together with an equally timely and combined therapeutic approach, is expected to result in greater treatment effectiveness, reduced damage accrual, and more comprehensive disease control, ultimately improving the overall quality of life.

In this study, SpA diagnosis was ultimately excluded in more than 80% of patients presenting with suspected MSK symptoms. These findings are consistent with evidence from another study involving a cohort of more than 1,000 psoriatic patients, in which, of the 293 patients initially classified as having suspected PsA, only 14% received a confirmed diagnosis, while the remaining 86% were assigned alternative diagnoses or categorized as having non-specific arthralgias (27).

As expected, based on the epidemiology of rheumatologic diseases, osteoarthritis and fibromyalgia emerged as the most common differential diagnoses, followed by other immune-mediated conditions, such as rheumatoid arthritis and connective-tissue diseases, not typically associated with IBD. Although the high prevalence of osteoarthritis and fibromyalgia is expected, their distinction from IBD-SpA remains complex and requires specific rheumatologic expertise, particularly because these conditions do not exclude the presence of IBD-related SpA. In this context, the challenge of distinguishing inflammatory enthesitis from fibromyalgia-related pain is particularly emblematic, with US playing a key role in confirming or excluding the inflammatory nature of the enthesis insertion sites (28). In addition to physical examination, MSK US has been demonstrated to have added value in supporting the differential diagnosis of conditions such as microcrystalline arthropathies and certain cases of osteoarthritis.

As an additional aspect of the differential diagnosis of SpA in IBD, a substantial subgroup of IBD patients with non-specific arthralgia, accounting for one-quarter of the suspected cases, was identified. In this regard, a key challenge in the study cohort was distinguishing non-specific arthralgia from fibromyalgia, which was primarily based on clinical judgment (including pain localization, distribution, and associated symptoms and their features) and on the lack of fulfilment of established classification criteria (20, 24). To the best of our knowledge, this is one of the first studies to describe this intriguing clinical entity in IBD patients, although it is currently one of the major research topics in patients with psoriasis. Indeed, up to 80% of new-onset PsA cases are preceded in the 2 years prior to diagnosis by a phase of non-specific arthralgia that confers a hazard ratio of 11 for progression in psoriatic patients (29). This has led PsA experts to believe that arthralgias may represent a prodromal or subclinical phase in the transition from psoriasis to PsA and may represent the target for interception strategies (30, 31). The demonstration of a significant proportion of patients with non-specific arthralgia among individuals with IBD lays the groundwork for further research into their potential predictive value for SpA and into opportunities for early interception. In this perspective, meaningful insights may emerge from the longitudinal follow-up of the DIAMANTE project, of which this study represents a cross-sectional phase analysis.

The complexity of early and accurate diagnosis of SpA in patients with IBD, and the consequent need for close collaboration between rheumatologists and gastroenterologists is further emphasized by the association analysis, in which the only independent predictor of a SpA diagnosis was the Swollen Joint Count (SJC), as assessed by the rheumatologist and not self-reported by the patients. This underscores the role of screening instruments, such as the DETAIL questionnaire, as useful first-level tools for identifying patients with IBD-SpA. However, a positive DETAIL questionnaire should not be considered diagnostic or used to guide therapeutic decisions in the absence of specialist evaluation. In addition, elevated FC levels showed a trend toward an association with a newly established SpA diagnosis. Although this finding is biologically plausible—given its well-recognized correlation with intestinal disease activity, which in turn is often associated with arthritis activity (3), further specifically designed studies incorporating comprehensive disease activity measures are warranted to validate these results and to explore the potential role of fecal and serum calprotectin as reliable biomarkers also in the joint domain of IBD-SpA. Finally, in the association analysis, vedolizumab—previously reported as a potential trigger of drug-induced arthritis (32)—was not associated with an increased risk in the study cohort. This is consistent with the results of a recent meta-analysis (33) and suggests that the observed association may reflect limited efficacy in preventing and treating one of the most frequent extraintestinal manifestations of IBD rather than a true drug-induced arthritis.

This study has some limitations. First, being conducted at a single tertiary gastroenterology centre may limit its generalizability, particularly regarding the broader applicability of a shared gastro–rheumatology clinic model. Second, the cross-sectional design of the analysis prevents the evaluation of the long-term outcomes of the gastro–rheumatology collaboration protocol, including the impact on the early identification of new IBD-SpA cases and on disease-related outcomes. Finally, this study examined the impact of the gastro–rheumatology collaborative model, with particular emphasis on detecting previously unrecognized cases of IBD-SpA in IBD patients. Furthermore, specific analyses may be required to evaluate outcomes in terms of IBD identification in SpA patients.

In conclusion, this study highlights how a multidisciplinary approach can help address the challenge of early and differential diagnosis of IBD-SpA, improving the sensitivity of its recognition while preventing the misclassification of MSK symptoms and thereby reducing the risk of overdiagnosis and unnecessary therapeutic changes.

## Data Availability

The raw data supporting the conclusions of this article will be made available by the authors, with reasonable request.
