# Supplementary material for: The role of a multidisciplinary approach in the early and differential diagnosis of inflammatory bowel disease–related spondyloarthritis: insights from a cross-sectional study
Source: Front Med (Lausanne). 2026 May 12;13:1824694. doi: 10.3389/fmed.2026.1824694 (PMC13201186; doi:10.3389/fmed.2026.1824694)
Supplement: Supplementary file 1 [file Table_1.docx]

Supplementary Material

| **Supplementary table 1**. Characteristics of patients with previous SpA diagnosis | |
| --- | --- |
|  | (n = 81) |
| **Males**, n (%) | 37 (45.7%) |
| **Age**, mean (SD) yrs | 52.5 (13.0) |
| **BMI**, mean (SD) | 24.3 (4.6) |
| **IBD duration**, mean (SD) yrs | 18.5 (10.7) |
| **IBD classification** |  |
| UC, n (%) | 29 (35.8) |
| CD, n (%) | 49 (60.5) |
| Other, n (%) | 3 (3.7%) |
| **SpA disease duration**, mean (SD) yrs | 14.6 (11.7) |
| **SpA diagnostic delay**, mean (SD) yrs |  |
| **SpA chronologic relationship** |  |
| SpA after IBD, n (%) | 35 (43.2%) |
| IBD after SpA, n (%) | 28 (34.6%) |
| Simultaneously, n (%) | 18 (22.2%) |
| **SpA pattern** |  |
| Peripheral arthritis, n (%) | 49 (60.5%) |
| Oligoarthritis, n (%) | 43 (54.4%) |
| Polyarthritis, n (%) | 36 (45.6%) |
| Axial involvement, n (%) | 46 (56.8%) |
| Dactylitis, n (%) | 26 (32.5%) |
| **CALF >200**, n (%) | 13 (22.2%) |
| **DETAIL score** |  |
| **Current treatment** |  |
| GC | 4 (5.5%) |
| Conventional immunosuppressant |  |
| SLZ | 6 (7.4%) |
| AZA | 1 (1.2%) |
| MTX | 4 (4.9%) |
| Biological Immunosuppressant | 60 (75.0%) |
| TNFi | 43 (53.1%) |
| IL12/23i and IL23i | 8 (9.9%) |
| VDZ | 2 (2.5%) |
| JAKi | 7 (8.6%) |
| SD, standard deviation. BMI, body mass index. IBD, inflammatory bowel disease. SpA, spondyloarthritis. DETAIL, Detection of Arthritis in Inflammatory bowel diseases questionnaire. adjOR, adjusted odds ratio. CI, confidence interval. | |
